# Cost-consequence analysis of surgical and clinical treatment modalities of laryngeal cancer

**DOI:** 10.1016/j.clinsp.2025.100585

**Published:** 2025-04-23

**Authors:** Alexandre Bezerra dos Santos, Patrícia Coelho de Soárez, Rossana Veronica Mendoza Lopez, Luciana Martins Rozman, Alessandro Gonçalves Campolina

**Affiliations:** a Departamento de Cirurgia de Cabeça e Pescoço, Instituto do Câncer do Estado de São Paulo, Faculdade de Medicina da Universidade de São Paulo (FMUSP), São Paulo, SP, Brazil; b Departamento de Medicina Preventiva, Faculdade de Medicina da Universidade de São Paulo (FMUSP), São Paulo, SP, Brazil; c Centro de Investigação Translacional em Oncologia, Instituto do Câncer do Estado de São Paulo, Faculdade de Medicina da Universidade de São Paulo (FMUSP), São Paulo, SP, Brazil

**Keywords:** Oropharyngeal neoplasms, Laryngeal neoplasms, Costs, Cost analysis

## Abstract

**Background:**

Laryngeal Squamous Cell Carcinoma (LSCC) may be treated clinically or surgically as a therapeutic option with a curative intention. The aim of this study is to compare direct medical costs and overall survival associated with the treatment of LSCC.

**Methods:**

Retrospective cost-consequence analysis, from the perspective of a Brazilian public hospital that included patients with LSCC, from 2014 to 2017. Unit costs were estimated using a macro-costing approach. The Propensity Score Matching method was used. Survival analyses were performed using the Kaplan-Meier method.

**Results:**

The therapeutic modalities were similar in terms of total costs: USD 32,259.65 for the clinical group and USD 34,385.87 for the surgical group (p = 0.215). Patients in the surgical group showed better overall survival than the clinical group (HR 0.53; p = 0.047).

**Conclusion:**

Both therapeutic modalities for the treatment of LSCC showed similar total costs. Nevertheless, overall survival was better in the surgical group.

## Highlights

• Laryngeal carcinoma may be treated clinically or surgically as an initial therapeutic option.
• Differences in terms of costs and outcomes has not been evaluated in Brazil.
• Clinical and surgical therapeutic modalities for laryngeal carcinoma showed similar total costs.
• Overall survival of laryngeal carcinoma patients was better in the surgical group.
• Further studies should consider implementing cost-effectiveness analysis.

## Introduction

Head and neck cancer is the sixth most common tumor in the world, with Squamous Cell Carcinoma (SCC) responsible for approximately 90 % of all histological types.^1^ The main risk factors are smoking and alcohol consumption;^2^ However, in recent years, the role of Human Papillomavirus (HPV) infection has been widely recognized as an important independent carcinogen.^3^

Head and neck cancers are responsible for a substantial proportion of health expenditures.^4^ The high costs associated with the treatment of these conditions are related to the multiplicity of affected anatomical sites, the large number of specialists involved in patient care, and the variety of available diagnostic and therapeutic modalities.^5^ The more advanced the tumors in their initial presentation, the greater the costs, given the need for more complex surgical procedures, intensive care, and a combination of therapeutic and rehabilitation modalities. Lower quality of life and worse survival results can also occur in advanced clinical stages.^5^

In developing countries, limited health resources make alternative diagnostic approaches to the management of these diseases highly relevant for the formulation of public policies at different levels: prevention, detection, treatment, rehabilitation, and palliative care.^6^^,^^7^ In these countries, whether due to a lack of information or the difficulty in accessing qualified medical care promptly, most head and neck tumors are diagnosed in advanced stages.^8^

Tumors of the larynx, which have historically been treated with surgery followed by Radiation Therapy (RDT), underwent a radical change in first-line treatment in the last two decades,^9^ with the introduction of the “clinical” modality ‒ combined treatment of Chemotherapy (QT) with RDT with curative intent.^10^, ^11^, ^12^ The similar initial results of the clinical and surgical modalities for the treatment of laryngeal Squamous Cell Carcinoma (LSCC) have given the clinical modality the name of “Organ Preservation”, as it offers the possibility of cure without the total laryngectomy and all the physical and psychosocial consequences involved.^10^^,^^11^^,^^13^ However, there seems to be a slight superiority in cure rates for the surgical treatment of advanced tumors, although the morbidity associated with the complete resection of the larynx, along with the occasional definitive tracheostomy, is still a relevant concern in clinical decision-making shared with patients.^14^

LSCC is prevalent in Brazil and shares the possibility of clinical or surgical treatment options, with many factors influencing the decision-making process, such as the possibility of mutilating procedures and resultant poor quality of life, breathing and swallowing difficulties, patient preferences, a good understanding of different options, and the social and economic impacts of the treatment.^11^^,^^13^^,^^14^ Therefore, for a comprehensive assessment of the global impact of LSCC and related therapeutic approaches, in addition to health outcomes, there is a need to assess economic outcomes, including the resources consumed as a result of the disease and its treatments.^15^^,^^16^

Unfortunately, studies on the cost-effectiveness of therapeutic modalities for LSCC are not widely available, and the use of resources and costs associated with healthcare for LSCC has not yet been investigated in Brazil. The aim of this study is to compare Overall Survival (OS) and direct medical costs associated with the treatment of LSCC using clinical or surgical treatment modalities at a Brazilian Cancer Center from 2014 to 2017.

## Methods

This was a retrospective cost-consequence analysis study aimed at estimating and comparing the overall, resource utilization, and costs associated with the treatment of LSCC, from the perspective of the *Instituto do Câncer do Estado de São Paulo* (ICESP), a public Brazilian health institution specialized in oncology. This study adopted an economic evaluation design, based on an observational study (historical cohort) with at least two years of follow-up to estimate the expended resources, costs, and clinical outcomes from January 2014 to December 2017.

### Study population

This study included patients over the age of 18-years, with LSCC, under treatment at the ICESP services of Head and Neck Surgery, Oncology, and Radiotherapy, who did not present contraindications to cancer treatment, whether clinical or surgical. Patients with a history of other synchronous malignancies, no curative intent (due to a lack of clinical conditions or treatment refusals), and those whose treatment was not fully performed at ICESP facilities were excluded. Palliative cases were also excluded.

### Setting and location

ICESP is the largest oncology hospital in the Brazilian public health system, located in São Paulo, the largest city in Brazil. It is a public hospital run by a non-profit private sector institution that works in a formal partnership with the state of São Paulo, collaborating to consolidate the Brazilian public health system, known as the Unified Health System (SUS). Since 2009, it has offered health services through a multidisciplinary team, organized for outpatient, inpatient, and hospice care.

ICESP has a building measuring 82,000 square meters, spread over 28 floors, where resources are allocated for oncological treatment. These resources include a chemotherapy infusion center with more than one hundred positions, six linear accelerators, seven computed tomography devices, four MRI machines, two PET-CTs, one SPECT, and other medical equipment. The hospital has 216 beds for clinical admissions and 150 beds for surgical admissions. Annually, around 18,000 hospital admissions; 213,000 medical consultations; 39,600 chemotherapy sessions, and 43,400 radiotherapy sessions are conducted.

Despite the fact that, according to the Brazilian constitution, the entire population would have access to the institution, most patients treated at ICESP belong to a lower socioeconomic stratum, without access to the private health system. The payment system is entirely derived from public resources – SUS.

### Strategies being compared

This study compared two different treatment strategies for LSCC: surgical treatment (total or partial laryngectomy) and non-surgical treatment (isolated radiotherapy, radiotherapy concurrently with chemotherapy, or induction chemotherapy followed by radiotherapy and chemotherapy). Partial laryngectomy or total laryngectomy could be followed by adjuvant radiation therapy. Concurrent chemoradiotherapy was performed with single-agent cisplatin or courses of cisplatin plus fluorouracil.

### Measurement and valuation of consequences

The primary endpoint of the study was to compare OS (measured from the day of diagnosis until death or last follow-up) for surgical and clinical treatments. The authors retrospectively evaluated consecutive patients with LSCC who received treatment from January 2014 to December 2017 at ICESP through a review of medical records. The methods used for the survival analysis are described below.

### Measurement and valuation of resources and costs

The cost estimate was carried out in three stages: identification of the relevant costs, measurement of expended resources, and valuation of resources.^4^ The process of identifying costing items began with a review of the literature on head and neck cancer cost studies and a panel of specialists. Since the perspective adopted was that of the healthcare provider, only the costs of the formal healthcare sector were included: medical and non-medical appointments, dental procedures, hospital admissions, visits to the emergency room, Intensive Care Unit (ICU) stay, surgeries, chemotherapy, radiation therapy, and imaging exams, including Computed Tomography (CT), Magnetic Resonance Imaging (MRI), and Positron Emission Tomography (PET-CT scan).

The unit costs were estimated by a macro-costing top-down methodology. To estimate the total cost of therapeutic modalities, patients were categorized according to the treatment options: clinical and surgical. The unit cost values were collected from the ICESP administrative database and cost estimates were presented in an aggregated and disaggregated manner, indicating the percentage of participation of each costing item. ICESP uses the absorption costing system, which makes it possible to calculate the cost by service units and by procedures, as well as to standardize the information and costs of the hospital units. This system enables the full appropriation of all direct costs to the product or service provided. This method is used by the production centers, where the cost centers are apportioned to define the real value of the cost of production. The valuation was based on reference values related to current practices by health services that are part of SUS in the State of São Paulo (non-profit organizations and direct management), considering the values in the current national currency (reais ‒ 2017).

The evaluation period was from January 2014 to December 2017, with a minimum time horizon of two years of follow-up of the cohorts. Costs were assumed to be similar for corrected values from July 2017, the middle of the last year of evaluation, to November 2021, when the analysis was conducted. The conversion rate was based on IGPM (Brazilian General Price Index for the Market) according to an online calculator,^17^ reaching an index of 1.26. Values were later converted into international dollars using the 2021 Purchasing Power Parity (PPP) exchange rate (2.162) calculated by a free web-based tool.^18^ An annual discount rate of 5 % was applied to the cost estimates, as recommended by the National Guidelines for Economic Health Assessment.^19^

The data sources for socio-demographic, clinical, and economic data were electronic medical records and ICESP administrative databases: Tasy® (electronic patient record system; access to demographic, clinical data, outcomes and number of resources used); HCMed® (visualization system for laboratory and imaging exams); Mosaic® (radiotherapy scheduling system); ICESP Cancer Registry, and Hospital-based records with systematic collection of clinical data from patients treated and/or diagnosed at the hospital.

### Survival analysis

The Propensity Score Matching (PSM) method was used to balance the two treatment groups based on the choice of variables associated with the treatments. The propensity scores were calculated by binary logistic regression, considering as an event of interest the patient having undergone surgery (P [patient underwent surgery]) The pairs were formed by the proximity of the calculated probabilities. The subjects were matched 1:1 according to the following covariates: comorbidities, Body Mass Index (BMI), and clinical staging. Finally, paired data were evaluated for survival. Overall survival was calculated in months from the date of diagnosis to the date of death or last follow-up. Patients alive or who missed their follow-up were considered censored.

### Statistical analysis and ethics

Patient characteristics were presented through frequencies and percentages for qualitative variables; and for quantitative variables, the mean, median, and standard deviation, along with first and third quartiles, were used. The association between qualitative variables was assessed by Pearson's Chi-Square test or Fisher's exact test. Comparison between groups for a quantitative variable was evaluated by the Mann-Whitney test. The survival curve was constructed using the Kaplan-Meier method for each of the treatments (clinical or surgical) and compared by the log-rank test. The significance level adopted was 5 %. Analyses were performed using the statistical software SPSS for Windows v.25.

The project was submitted to the ICESP Research Center and, after approval, was sent to the ICESP Research Ethics Committee, following Resolution 466/2012 of the National Health Council of the Ministry of Health (CONEP / MS). The Research Ethics Committee approved the study on 11/18/2019, n° 1600. The preparation of the manuscript followed the Consolidated Health Economic Evaluation Reporting Standards 2022 (CHEERS 2022) Statement.

## Results

From January 2015 to December 2017, a total of 834 patients with LSCC were admitted and treated at ICESP. After applying the exclusion criteria, 257 patients with LSCC (141 clinical and 116 surgical) were included in the study.

Patients with LSCC had a mean age of 59.4 years for the clinical group and 60.8 for the surgical group. The majority were male (91.5 % in the clinical and 87.9 % in the surgical group), white (74.8 % in the clinical and 78.4 % in the surgical group), married (56.9 % in the clinical and 67.9 % in the surgical group), tobacco users (70.9 % in the clinical and 56.9 % in the surgical group), and alcohol users (51.8 % in the clinical group). A significant proportion of patients had a primary and/or high school education; 46.2 % and 40.4 % in the clinical group, as compared to 43.3 % and 40.3 % in the surgical group (Table 1).

**Table 1:** Sociodemographic and clinical characteristics of patients with Larynx SCC, ICESP/SP, 2014 to 2017.

Characteristics	Clinical group (n = 141)	Surgical group (n = 116)	p^a^
*Mean age, years, (SD)*	59.4 (9.7)	60.8 (9.4)	0.347
*Gender, n (%)*			
Male	129(91.5)	102 (87.9)	
Female	12 (8.5)	14 (2.1)	
*Race (%)*			0.233
White	104 (74.8)	91 (78.4)	
Brown	30 (21.6)	25 (21.6)	
Black	4 (2.9)	0 (0)	
Yellow	1 (0.7)	0 (0)	
*Marital Status (%)*			0.343
Single	27 (19.7)	18 (16.1)	
Married	78 (56.9)	78 (67.9)	
Divorced	19 (13.9)	10 (8.9)	
Widowed	13 (9.5)	8 (7.1)	
*Level of Education (%)*			0.568
Illiterate	2 (1.9)	4 (6.0)	
Primary school	48 (46.2)	29 (43.3)	
High School	42 (40.4)	27 (40.3)	
Higher Education	12 (11.5)	7 (10.4)	
*Tobacco use (%)*			0.043
No	6 (4.3)	11 (9.5)	
Former	35 (24.8)	33.6 (66)	
Yes	100 (70.9)	66 (56.9)	
*Alcohol use (%)*			0.035
No	18 (12.8)	29 (25)	
Former	50 (35.5)	32 (27.6)	
Yes	73 (51.8)	55 (47.4)	
*Comorbidities (%)*			0.001
No	88 (62.9)	48 (42.5)	
Yes	52 (37.1)	65 (57.5)	
*Karnofsky scale (%)*			0.242
30	0 (0)	0 (0)	
40	1 (0.7)	1 (0.9)	
50	7 (5.1)	1 (0.9)	
60	3 (2.2)	3 (2.7)	
70	8 (5.8)	1 (9.8)	
80	27 (19.7)	14 (12.5)	
90	68 (49.6)	66 (58.9)	
100	23 (16.8)	16 (14.3)	
*ECOG (%)*			0.371
0	36 (26.1)	38 (33.6)	
1	74 (53.6)	57 (50.4)	
2	16 (11.6)	12 (10.6)	
3	12 (8.7)	5 (4.4)	
4	0 (0)	1 (0.9)	
*Stage (%)*			0.110
I	15 (10.6)	16 (13.8)	
II	7 (5.0)	10 (8.6)	
III	26 (18.4)	26 (22.4)	
IVa	37 (26.2)	36 (31.0)	
IVb	56 (39.7)	28 (24.1)	

SCC, Squamous Cell Carcinoma; ECOG, Eastern Cooperative Oncology Group.

a Chi-Squared test.

In the clinical group, most patients were classified as having no comorbidities (62.9 %), while in the surgical group, only 42.5 % of the patients had no comorbidities. According to the Eastern Cooperative Oncology Group (ECOG) scale, 8.7 % of the patients in the clinical group had a performance status of ≥ 3, whereas 5.3 % of the patients in the surgical group had a performance status of ≥ 3. The Karnofsky scale indicated that 49.6 % of the patients in the clinical group had a performance of 90 %, while 58.9 % of patients in the surgical group showed the same performance. Most patients in the clinical group were classified as stage IVb (39.7 %), while those in the surgical group were mostly classified as stage Iva (31.0 %).

For patients in the clinical group, the median cost per patient treated was U$32,259.65 (SD U$16,841.10), whereas, for patients in the surgical group, the median cost was U$34,385.87 (SD U$17,750.56), as shown in Table 2. The cost items that differed between the clinical and surgical groups included dental procedures, surgeries, chemotherapy, radiation therapy, emergency room, ICU, and length of stay (Table 2). Patients in the clinical group incurred higher costs for dental procedures, chemotherapy, radiation therapy, emergency room visits, and length of stay.

**Table 2:** Health resources, total and mean cost per patient (U$), according to cancer treatment modality, ICESP, São Paulo, 2014 to 2017.

	Clinical (n = 141)		Surgical (n = 116)		p (Use)	p (Cost)
Item	Use, Mean (SD)	Cost, Mean (SD)	Use, Mean (SD)	Cost, Mean (SD)		
Medical Cons.	32.7 (18.5)	4197.71 (2366.86)	33.3 (17.2)	4200.79 (2177.32)	0.645	0.786
MultiP Cons.	31.5 (19.7)	849.97 (635.67)	27.8 (14.1)	704.69 (493.67)	0.320	0.080
Dental procedure	19.1 (17.2)	1951.56 (1964.30)	10.4 (11.7)	1041.31 (1254.57)	<0.001	<0.001
Surgeries	0.7 (1.0)	4172.84 (7030.73)	1.9 (1.2)	11,290.56 (11,539.91)	<0.001	<0.001
CTX	4.1 (4.4)	1910.85 (2139.62)	1.3 (2.2)	628.72 (1035.18)	<0.001	<0.001
RT	33.0 (5.3)	4930.00 (708.80)	26.3 (14.6)	3914.70 (2172.87)	0.003	0.002
ER	5.9 (5.4)	8016.72 (8332.48)	4.4 (4.3)	6104.81 (6899.70)	0.026	0.032
ICU	0.4 (0.7)	879.78 (1709.06)	0.9 (1.3)	1627.60 (2223.46)	<0.001	<0.001
LOS	2.9 (2.9)	4093.00 (5722.39)	3.1 (2.0)	3867.04 (4740.53)	0.040	0.134
CT	11.3 (14.0)	898.41 (1162.43)	9.4 (5.1)	775.57 (415.65)	0.998	0.540
MRI	0.5 (1.3)	81.43 (212.26)	0.4 (1.0)	59.36 (163.67)	0.232	0.198
PET-CT scan	0.1 (0.4)	277.38 (829.06)	0.1 (0.3)	170.72 (608.42)	0.396	0.357
Total		32,259.65 (16,841.10)		34,385.87 (17,705.56)		0.215

Medical Cons., Doctor's Appointment; MultiP Cons., Multidisciplinary Appointment; CTX, Chemotherapy; RT, Radiation Therapy; ER, Emergency Room; ICU, Intensive Care Unit; LOS, Length of stay in hospitals; CT, Computerized Tomography, MRI, Magnetic Resonance Imaging.

**Fig. 1:**
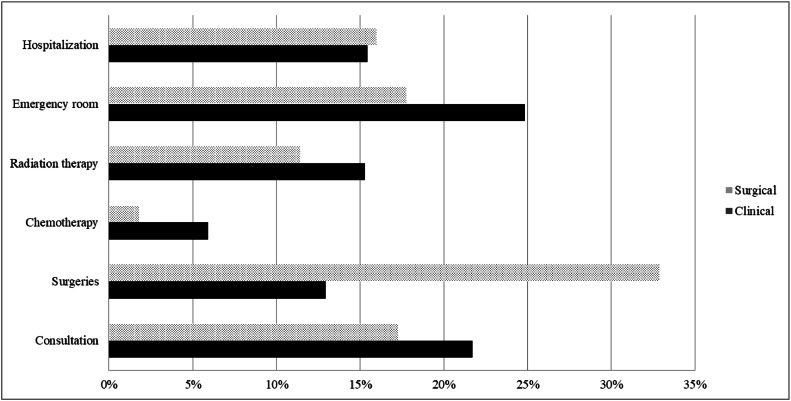
Proportional costs of the clinical and surgical treatments of LSCC, ICESP, São Paulo, 2014 to 2017.

Fig. 1 illustrates the items that had the greatest impact on the total cost for both patient groups. In the clinical group, the items with the most significant impact on the total cost were emergency room (25 %), consultation (22 %), radiation therapy (15 %), and hospitalization (15 %). In the surgical group, the items with the most significant impact on the total cost were surgeries (33 %), emergency room (18 %), doctor's appointments (17 %), and hospitalization (16 %).

**Fig. 2:**
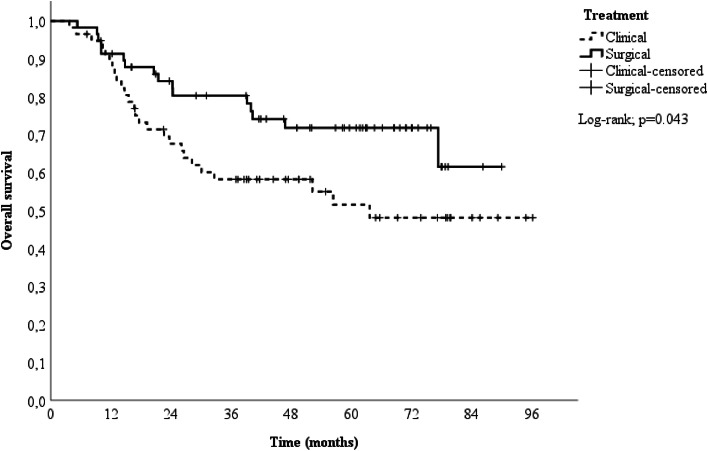
LSCC Clinical and Surgical Survival Curves, ICESP, São Paulo, 2014 to 2017.

A 1:1 propensity score-matched analysis was performed, resulting in a total of 116 patients, 58 in the surgical group and 58 in the clinical group. After a median follow-up time of 51.9-months (range 5.3‒90.1 months) in the surgical group and 38 months (range 3.8‒96.2 months) in the clinical group, the two-year OS differed between the groups (84.2 % vs. 67.7 % for the surgical and clinical groups, respectively), as shown in Fig. 2. Patients in the surgical group had a better survival rate than those in the clinical group (HR = 0.53; 95 % CI 0.28 to 0.99; p = 0.047).

## Discussion

In low- and middle-income countries, the potential benefits of cost analysis studies for managing LSCC are recognized; however, the literature on economic outcomes and cost comparisons between clinical and surgical treatment approaches is limited and inconsistent. This is primarily due to the heterogeneity of anatomical sites involved, the multidisciplinary nature of healthcare teams, and the variability in treatment practices across different regions.^20^

This study contributes to the existing literature by demonstrating that, within the Brazilian context, there were no significant differences in total costs between clinical and surgical treatments for LSCC, despite observing different overall survival outcomes between the two approaches. These findings contrast with existing literature, likely due to methodological differences among studies, many of which focus on specific treatment modalities rather than broad clinical or surgical approaches.

For instance, in laryngeal cancer, Diaz-de-Cerio et al. found that surgical costs were higher than those for radiotherapy (with or without chemotherapy) when comparing laryngo fissure cordectomy with radiotherapy.^21^ Similarly, in a Dutch study on oropharynx cancer, the number of inpatient days made the surgical costs higher than radiotherapy costs,^22^ while in a German study, it was found that higher costs for oropharynx surgical patients were acceptable because of better clinical outcomes.^23^ However, these studies predominantly included early-stage cases (near 50 % T2 and 25 % T1 in the German study), a scenario quite different from this study, where the clinical cohort included 65.9 % T4 tumors, and the surgical cohort included 55.1 % T4 tumors.

Unfortunately, most cases in this study were diagnosed at advanced clinical stages, often with limited curative options. A significant portion of patients also had comorbidities and high functional impairment, as indicated by the ECOG scale and Karnofsky performance index. The mild predominance of clinically advanced cases is primarily attributed to patients’ preference for non-surgical treatment, aiming to avoid the reduced quality of life associated with surgical procedures. However, even in the surgical cohort, the complexity of cases, particularly those involving free-flap reconstruction, led to higher costs, both due to the surgery itself and the prolonged ICU stays, as observed in other studies.^24^^,^^25^

The greatest impact on total costs was associated with hospitalization, particularly in the surgical cohort, where it accounted for approximately 70 % of the costs, consistent with other studies. Hospitalization costs showed a slight predominance, comprising between 50 % and 60 % of the total costs. This was influenced by several factors, including dental procedures (due to poor dental health in the Brazilian population), radiation therapy, and frequent doctor's appointments, particularly for rehabilitation*.* Unlike other studies, outpatient chemotherapy costs were low,^26^ mainly because the first-line anticancer drugs used in the public institution are based on carboplatin and paclitaxel. Newer, more expensive drugs are not yet reimbursed by the Brazilian health system and are currently used only in clinical trials for treating recurrences; hence, they were excluded from this analysis.

Regarding hospitalization costs, a significant impact was observed due to emergency room visits, driven by various factors such as side effects of chemotherapy or chemoradiation therapy, surgical complications like fistulas, issues with catheters or tracheostomies, infections, or blood loss. A previous Brazilian study showed higher costs in more advanced laryngeal cancers,^27^ although costs were lower in other advanced tobacco-related cancers, such as esophageal and pulmonary cancers, in very advanced cases due to a lack of therapeutic options. Generally, previous studies indicated higher costs in more advanced head and neck tumors,^28^ highlighting the potential economic benefits of early detection programs.^29^

Survival analysis showed better outcomes in the surgical group, suggesting greater effectiveness, as observed in another study,^30^ comparing surgical and organ preservation approaches in advanced cases. Davis et al. similarly found higher direct costs in organ preservation treatments for advanced laryngeal cases but with equivalent overall survival.^31^

This study has several limitations that should be noted: 1) This research was conducted from the perspective of a reference center in the largest Brazilian city, which may not reflect the broader national reality regarding health service provision and costs, limiting the generalizability of the results; 2) This is the first comprehensive study on LSCC treatment at ICESP, and further analyses, such as population sub-stratification by stage or subsite, are necessary; 3) As with most studies on head and neck cancer, only direct costs were analyzed, despite evidence that indirect costs, particularly related to substantial work loss, have a significant impact, as demonstrated in case series from the USA^32^ and Norway;^33^ and 4) In this cohort, HPV-testing was performed only in selected cases, and it was not considered in the analyses. HPV-related oropharyngeal cancer incidence is higher in Brazilian private healthcare hospitals, particularly in younger patients,^34^ but this incidence is less significant in public hospitals. At ICESP, the incidence was 17.7 % in oropharyngeal cases and 14.3 % in LSCC, suggesting that HPV status likely had minimal impact on total costs.^35^

Despite these limitations, this is the first study, to our knowledge, to assess the resources and costs of LSCC treatment in a single quaternary oncological hospital in Brazil. These initial findings highlight the potential advantages of surgical treatment for advanced laryngeal cancer, but a better stratification by clinical stages is necessary. Further studies should also incorporate cost-effectiveness analysis to better understand the monetary value of different therapeutic strategies for LSCC, which could inform the allocation of scarce resources in oncological care.

## Conclusions

Clinical and surgical treatment modalities for LSCC showed similar total costs from the perspective of a Brazilian Cancer Center. However, overall survival was better in the surgical group.

## CRediT authorship contribution statement

**Alexandre Bezerra dos Santos:** Conceptualization, Methodology, Investigation, Writing – original draft. **Patrícia Coelho de Soárez:** Supervision, Validation, Writing – review & editing. **Rossana Veronica Mendoza Lopez:** Data curation, Investigation, Software, Formal analysis, Validation. **Luciana Martins Rozman:** Visualization, Investigation, Writing – review & editing. **Alessandro Gonçalves Campolina:** Project administration, Supervision, Methodology, Validation, Writing – review & editing.

## Declaration of competing interest

The authors declare no conflicts of interest.
